# Head and neck radiotherapy quality assurance conference for dedicated review of delineated targets and organs at risk: results of a prospective study

**DOI:** 10.1017/s1460396922000309

**Published:** 2022-11-04

**Authors:** J. C. Farris, N. B. Razavian, M. K. Farris, J. D. Ververs, B. A. Frizzell, C. M. Leyrer, L. F. Allen, K. M. Greven, R. T. Hughes

**Affiliations:** Department of Radiation Oncology, Wake Forest School of Medicine, Winston Salem, NC, USA

**Keywords:** quality assurance, peer review, head and neck, radiotherapy, radiation, chart rounds

## Abstract

**Purpose:**

Head and neck (HN) radiotherapy (RT) is complex, involving multiple target and organ at risk (OAR) structures delineated by the radiation oncologist. Site-agnostic peer review after RT plan completion is often inadequate for thorough review of these structures. In-depth review of RT contours is critical to maintain high-quality RT and optimal patient outcomes.

**Materials and Methods:**

In August 2020, the HN RT Quality Assurance Conference, a weekly teleconference that included at least one radiation oncology HN specialist, was activated at our institution. Targets and OARs were reviewed in detail prior to RT plan creation. A parallel implementation study recorded patient factors and outcomes of these reviews. A major change was any modification to the high-dose planning target volume (PTV) or the prescription dose/fractionation; a minor change was modification to the intermediate-dose PTV, low-dose PTV, or any OAR. We analysed the results of consecutive RT contour review in the first 20 months since its initiation.

**Results:**

A total of 208 patients treated by 8 providers were reviewed: 86·5% from the primary tertiary care hospital and 13·5% from regional practices. A major change was recommended in 14·4% and implemented in 25 of 30 cases (83·3%). A minor change was recommended in 17·3% and implemented in 32 of 36 cases (88·9%). A survey of participants found that all (*n* = 11) strongly agreed or agreed that the conference was useful.

**Conclusion:**

Dedicated review of RT targets/OARs with a HN subspecialist is associated with substantial rates of suggested and implemented modifications to the contours.

## Introduction

The American College of Radiology recommends that all radiation oncology departments perform routine peer review of treatment plans prior to delivery.^1^ Radiotherapy (RT) for head and neck (HN) cancers is often delivered using intensity-modulated radiotherapy or volumetric modulated arc therapy.^2^ As a result, HN RT plans are characterised by extremely complex radiation dose distributions to areas within the patient that are based entirely on target and organ at risk (OAR) structures delineated by the radiation oncologist. Routine departmental peer review often involves case discussion (approximately 1–5 min per patient) with brief review of the history, documentation and prescription with minimal time spent examining the actual structures on which the RT plan is based.^3^ As a result, these venues are not well-equipped to respect the high level of detail involved in the HN RT planning process. It is well-established that the quality of HN RT plans is strongly associated with survival.^4^

At our institution, patients are reviewed in a weekly ‘chart rounds’ conference that provides a board review of the overall treatment plan and intent.^5^ Prior to the initiation of the weekly HN RT Quality Assurance Conference (QAC), there was no standardised HN RT-specific peer review process—the RT plan was based on independently delineated target and OAR structures. In-depth reviews of individual structures with other radiation oncologists or radiology were performed on an ad hoc basis.

To improve RT plan quality and consistency, as well as create an additional venue to contribute to radiation oncology trainee education, we developed a weekly HN RT QAC. This facilitated a dedicated review of the target/OAR structures prior to RT plan finalisation/delivery for each HN cancer patient treated. The hypothesis was that this in-depth review of RT target and OAR volumes would result in clinically relevant rates of major and minor changes to these structures prior to RT plan finalisation by dosimetry. In this study, we assessed initial results of RT contour changes as a result of the implementation of the HN RT QAC.

## Materials and Methods

### Head and neck RT quality assurance conference

The HN RT QAC is a weekly conference intended to provide in-depth review of delineated target/OAR structures for all patients treated with HN RT. The QAC is entirely virtual, based in an academic institution, and available to affiliated community practice providers through virtual teleconferencing/screen share capabilities. The conference was routinely attended by 3–4 attending radiation oncologists, at least 1 with site-specific expertise in HN radiation oncology. While the directive was to review all cases for patients receiving RT with curative intent, a small proportion of more technically challenging (i.e., prior RT, complex extent of disease or involvement of critical OARs) cases of palliative RT were also allowed. Prior to presentation at the conference, all contours were reviewed by the treating physician. Thus, the contours reviewed by the group were considered to be the final version of the contours that were otherwise ready to be used for dosimetric planning if no changes were recommended during the QAC. The contour review was performed prior to RT plan creation. The structure of each individual case review (see Appendix A for Sample Agenda) includes presentation of the relevant clinical, pathologic and radiographic details; slice-by-slice on-screen display of gross/clinical/planning target volumes (PTVs), review of rationale for target/dose selection, display of OARs, review of the fused diagnostic imaging and consideration of other anatomic/dosimetric challenges as needed.

### Study population and outcomes

We analysed an Institutional Review Board-approved prospective registry of consecutive patients whose RT volumes were reviewed in the QAC between August 2020 and April 2022. The results of the peer review discussion including the number/nature of recommended RT volume modifications and whether these recommended changes were implemented were recorded. The rate of compliance was defined as the proportion of curative-intent RT volumes submitted to the HN RT QAC out of the total number of curative HN RT volumes delivered during the study period. This measure was only assessable for the main centre, as this was the primary site of the QAC.

**Figure 1.**
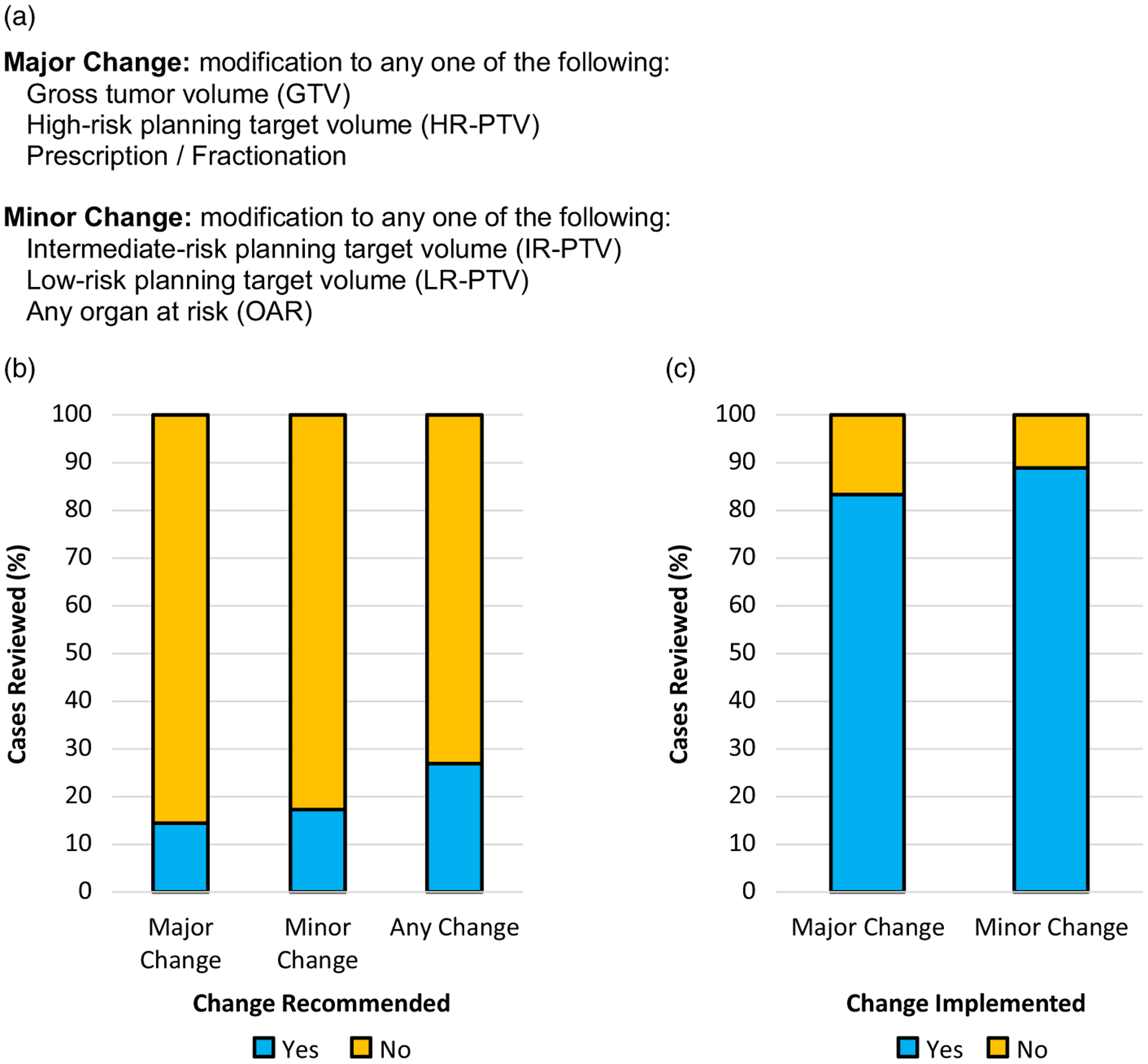
(a) Definitions of major and minor changes used for the prospective data collection of HN RT QAC cases. (b) Per cent of cases in which major or minor changes were suggested. (c) Per cent of cases in which major and minor changes were implemented by the treating physician.

The definitions of major and minor changes after case review are defined as previously described by prior studies in order to maintain consistency across studies within the growing body of literature on this topic (Figure 1A).^6,7^ During the conference, the RT volumes were discussed by the group and changes were recommended as needed. Recommended major changes were the responsibility of the treating physician to implement. If recommended, minor changes could be implemented at the discretion of the treating physician. Since contour review was to be completed prior to the completion of the dosimetric planning process, re-planning rates were not a relevant endpoint.

At the end of the study period, all participants (faculty physicians as well as resident trainees) that had been present for at least one QAC were surveyed regarding their agreement with the following statement: ‘I have found the weekly head and neck contour review conference to be helpful when delineating head and neck treatment volumes’. Degree of agreement was measured using a 5-point Likert scale. A score of 1 indicated strong disagreement, and a score of 5 indicated strong agreement.

Descriptive statistics were used to characterise the patient population. The rate of major RT volume changes recommended by the RT QA conference was calculated using summary statistics including count (frequency). Categorical variables including primary site, tumour and nodal stage and treatment intent (definitive, postoperative, or palliative) were tested for association with any change recommendation using bivariable tests (Fisher’s exact or chi-square where appropriate). Statistical significance was set at *p* < 0·05. All statistical analyses were performed using R version 3.6 (R Foundation for Statistical Computing, Vienna, Austria).

## Results

### Patient and treatment characteristics

Two-hundred and eight cases treated by 8 individual providers were reviewed at the HN RT QAC: 86·5% from main centre, 13·5% from one of three affiliated regional practices. Compliance to review at the main centre was 85% (170 of 200 total cases). Patient characteristics are summarised in Table 1. The most common primary sites were oropharynx (29·8%) and oral cavity (22·6%). The majority of patients had T3–4 disease (67·7%); nodal stage distribution was N0 (40·9%), N1 (22·1%), N2 (20·7%) and N3 (16·3%). RT intent was definitive (43·8%), postoperative (49·5%), preoperative (1·4%) or palliative (5·3%). Over the 86-week study period, an average of 2·4 cases per week were reviewed.

### Changes to treatment structures

**Figure 2.**
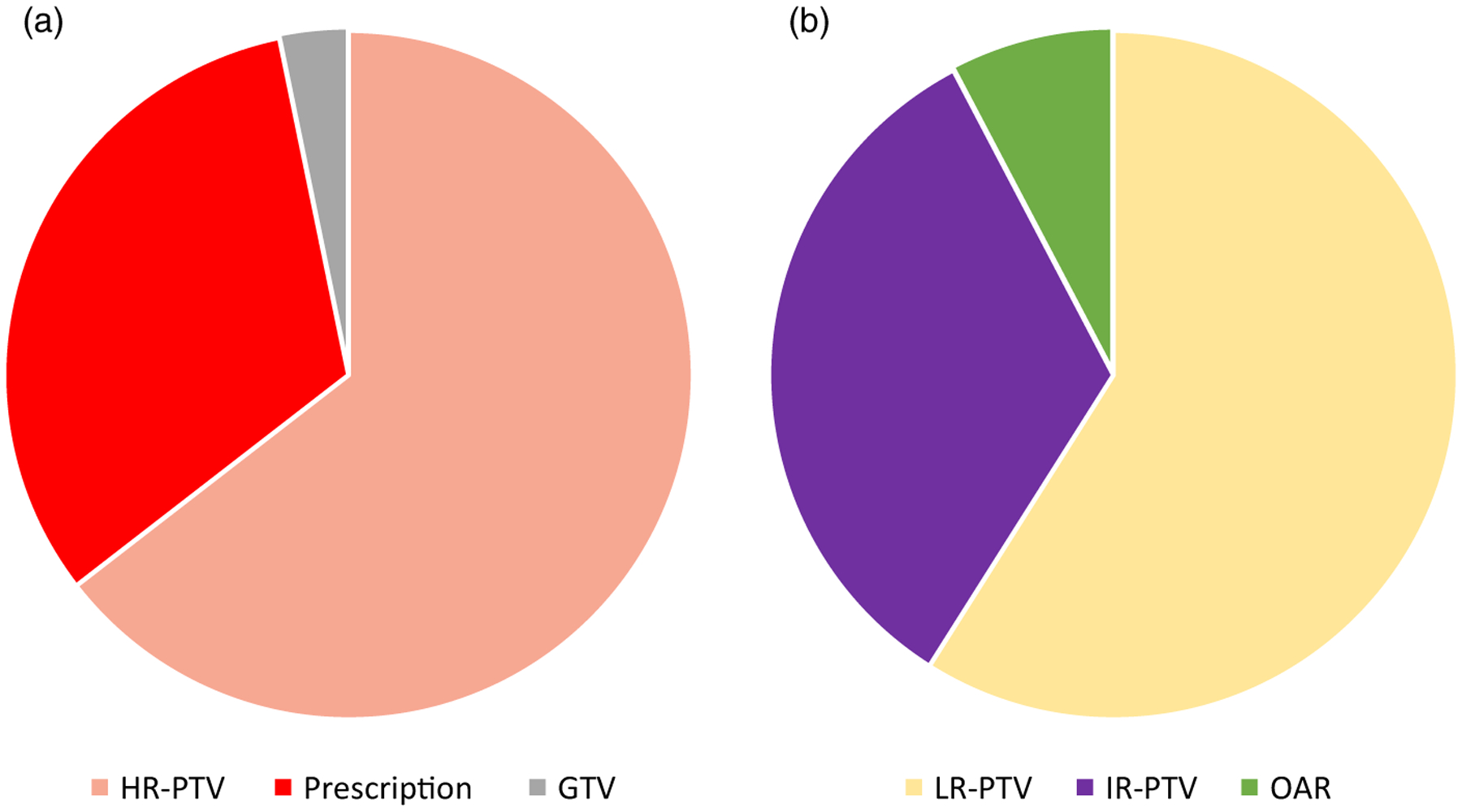
(a) Distribution of the types of major changes recorded. (b) Distribution of the types of minor changes recorded.

Changes recommended after review in the QAC are summarised in Figure 1B, with any change indicating either major, minor or both changes recommended. A recommendation for major change was made in 14·4%. Major changes were implemented in 25 of 30 cases (83·3%, Figure 1C). The distribution of major change types is depicted in Figure 2A and included (each case could have more than one recommended change) high-risk PTV (*n* = 20), RT prescription dose and/or fractionation (*n* = 10), and Gross Tumor Volume (GTV) (*n* = 1) (Figure 2A). A minor change was recommended in 17·3%. The distribution of minor change types (Figure 2B) included low-risk PTV (*n* = 23), intermediate-risk PTV (n = 13) and OAR (n = 3). Minor changes were implemented in 32 of 36 cases (88·9%) (Figure 1C). Additional workup was suggested in 3 cases (1·4%; 2 imaging studies and 1 procedure) and completed in two. No significant association between any change recommendation (yes versus no) and clinical factors such as primary site, T stage, N stage or treatment intent was identified. Correction of contour suggested changes was associated with a statistically significant delay of 1 day in overall treatment planning time from CT simulation to treatment start (*p* = 0·001).

### Participant-rated utility of a head and neck RT QA conference

The utility of the HN RT QAC was briefly assessed using a single measure of satisfaction scored on a 5-point Likert scale at the end of the study period. In response to the question of whether the weekly HN RT QAC was helpful when delineating HN treatment volumes, 9 of 11 participants strongly agreed, and 2 agreed. On a 5-point Likert scale, 5 representing ‘Strongly Agree’ and 1 representing ‘Strongly Disagree’, the mean participant response score was 4·8 (standard deviation, 0·40).

## Discussion

As complexity of HN RT has evolved, so has the need for more in-depth peer review to ensure optimal treatment outcomes. Routine physician peer review of superficial aspects of the overall RT plan detects as few as 55% of errors.^8^ The degree of detail with which the review is conducted varies among institutions, particularly with regard to visualising RT target/OAR structures. The point in the planning process at which cases are reviewed is also variable. Routine practice includes peer review after the planning process has already been finalised. If an issue is detected at this stage, work is duplicated to correct the issue; owing to this barrier, depending on the severity of the issue, the change may not be implemented at all. A study of multiple tumour sites found that post-dosimetry plan review was associated with fewer changes than pre-dosimetry review of contours, suggesting that review of the structures on which the RT plan is designed may facilitate optimised treatment, particularly in the HN site where complex, highly conformal modalities predominate.^9–11^

Various high-volume cancer centres have reported outcomes of a dedicated framework for HN RT quality assurance, keeping in mind that the impact of peer review has been suggested to be significant if rates of change exceed 10% for reviewed cases.^12^ Changes to the proposed treatment target or normal tissue volumes occur in approximately 14–55% of reviewed cases.^6,7,13^ In a multidisciplinary planning conference, 55% of cases reviewed resulted in changes, and 61% of these changes were clinically significant resulting in inclusion or exclusion of a distinct area or structure. These included important structures or regions such as gross tumour, postoperative bed, at-risk nodal basins, adjacent bone/skull base/perineural structures and OARs. Approximately 30% resulted in a change to either the gross tumour or clinical target volume contours. In our conference, we found that 14·4% of changes required major changes, while 17·3% required minor changes with high rates of provider implementation, consistent with prior studies. This provides further evidence that a dedicated review of contours results in a substantial proportion of clinically meaningful changes that would otherwise not occur without a platform for formal contour review. Additionally, the present study presents data indicating a high level of satisfaction of participating providers with the contour review process. Interestingly, our results did find a statistically significant delay of 1 day associated with suggestions for contour changes compared to those without suggested changes. While this represents a statistical difference, it is likely of small clinical relevance when weighted against the benefit of an improved treatment plan delivered to the patient.

Further exploration of this practice is warranted to more clearly define the rates of change as well as the clinical and dosimetric impacts of this change. The definition of major and minor change in the context of this study was determined ab initio to be consistent with prior reports of a similar nature using identical predefined criteria determining major versus minor change.^6,7^ Major changes included changes to any GTV, high-dose PTV or the prescribed dose and fractionation; minor changes included other changes to intermediate- or low-dose PTV or OARs. It is conceivable that this strict definition may mischaracterise changes with major clinical relevance as ‘minor changes’ (i.e., change to low-dose PTV to spare a critical structure, thus avoiding significant toxicity or modification of an OAR that impacts a clinically relevant dosimetric parameter). However, approaching this issue with a more subjective view of what constitutes ‘major’ and ‘minor’ changes also may limit reproducibility and generalisability of studies reporting these changes. It is clear that further analysis and attention to the major and minor classifications with regard to clinical impact and relevance is needed. Future aims of studies evaluating RT quality assurance initiatives such as this should consider these nuances and take steps to maintain objective classification with attention to the potentially variable clinical relevance of the categorisation scheme.

There are additional limitations to this study. The relatively small sample size limits power to evaluate differences in rates of RT volume changes across clinical factors. Additionally, despite overall favourable findings, the relatively small number of respondents in the participant-rated assessment of utility limits interpretability. There is also not a control group that would facilitate comparison in clinical outcomes or dosimetric changes with and without this dedicated RT contour review conference.

## Conclusions

The implementation of a dedicated HN RT QAC results in excellent rates of compliance and significant proportions of major and/or minor changes with a high degree of implementation. Further investigations to optimise this process, improve generalisability, facilitate widespread implementation and better define change outcomes of interest to align with clinically relevant outcomes are needed.

## Supplementary Material

Supplemental Material

## Figures and Tables

**Table 1.** Patient characteristics and treatment details.

	n (%)
Location Treated	
Main Campus	180 (86·5)
Regional Practice	28 (13·5)
Primary Site	
Oropharynx	62 (29·8)
Oral Cavity	47 (22·6)
Skin	33 (15·9)
Larynx	32 (15·4)
Nasal Cavity/Paranasal Sinus	11 (5·3)
Salivary	9 (4·3)
Nasopharynx	8 (3·8)
Hypopharynx	2 (2·0)
Unknown	1 (0·5)
Other	3 (1·4)
Histology	
Squamous Cell Carcinoma	179 (86·1)
Other	29 (13·9)
Tumour Stage	
T0	14 (6·7)
T1	19 (9·1)
T2	34 (16·3)
T3	62 (29·8)
T4	79 (38·0)
Nodal Stage	
N0	85 (40·9)
N1	46 (22·1)
N2	43 (20·7)
N3	34 (16·3)
Metastasis Stage	
M0	201 (96·6)
M1	7 (3·4)
Treatment Intent	
Neoadjuvant	3 (1·4)
Adjuvant	103 (49·5)
Definitive	91 (43·8)
Palliative	11 (5·3)

## Data Availability

Research data are stored in an institutional repository and will be shared upon request to the corresponding author.
