# Supplementary material for: Head and neck radiotherapy quality assurance conference for dedicated review of delineated targets and organs at risk: results of a prospective study
Source: J Radiother Pract. Author manuscript; Available in PMC 2024 Jan 30. (PMC10827337; doi:10.1017/s1460396922000309)
Supplement: Supplemental Material [file NIHMS1959857-supplement-Supplemental_Material.docx]

Data Supplement Appendix A: Sample Head and Neck Quality Assurance Conference Agenda

**Head and Neck Radiation Therapy Quality Assurance Conference**

**Location**: Webex Virtual Meeting (subject to change)

**Attendees**: Radiation Oncology (faculty and trainees), WFBH satellite practices

**Patients**: [those simulated in the weeks prior to meeting date]

**Information Presented**

- Medical record number
- Primary disease site
- AJCC 8^th^ Edition TNM Stage
- Indication: Definitive/Adjuvant/Neoadjuvant/Palliative
- Clinical examination findings, as indicated
- Staging imaging: CT, MR, PET/CT

**Discussion Order**

- Primary target
  - Primary tumor delineation
  - Selection of target and expansion
- Neck target
  - Grossly involved LN delineation, if applicable
  - Selection of LN levels to include in at-risk CTVs
  - Adherence to consensus guidelines: Y/N
- Prescription Dose/Fractionation
  - High-risk PTV
  - Intermediate-risk PTV, if applicable
  - Low-risk PTV, if applicable
- Image Guidance: PET, MR, CT
- Special considerations: nerve path for PNI, bolus, OSL, IMRT/VMAT v. 3D technique, etc

**Recommendations**

Definitions of Major/Minor Recommended Changes

- Major Change: a change to at least 1 of the following
  - GTV primary (GTVp)
  - GTV nodes (GTVn)
  - High-dose PTV
  - Prescribed dose and fractionation
- Minor Change: a change to at least 1 of the following
  - Intermediate-dose PTV
  - Low-dose PTV
  - Any organ at risk (OAR)
- Require re-plan or plan change (if already planned): Y/N
- Recommended Change Made: Y/N

**Learning Objectives**

- Time permitting, learning objectives assigned at the prior meeting will be discussed and new objectives set for next upcoming meeting. These may include topics including
  - Consensus guideline determination/discussions
  - Target and OAR definitions (basic or advanced, tailored to trainee level of training)
  - Neuroanatomy high-yield for HN RT considerations
